# Non-local mean denoising using multiple PET reconstructions

**DOI:** 10.1007/s12149-020-01550-y

**Published:** 2020-11-26

**Authors:** Hossein Arabi, Habib Zaidi

**Affiliations:** 1 Division of Nuclear Medicine and Molecular Imaging, Department of Medical Imaging, Geneva University Hospital, 1211 Geneva 4, Switzerland; 2 Geneva University Neurocenter, Geneva University, 1205 Geneva, Switzerland; 3 Department of Nuclear Medicine and Molecular Imaging, University of Groningen, University Medical Center Groningen, 9700 RB Groningen, The Netherlands; 4 Department of Nuclear Medicine, University of Southern Denmark, 5000 Odense, Denmark

**Keywords:** PET, Image quality, Non-local means, Filtering, Iterative reconstruction

## Abstract

**Objectives:**

Non-local mean (NLM) filtering has been broadly used for denoising of natural and medical images. The NLM filter relies on the redundant information, in the form of repeated patterns/textures, in the target image to discriminate the underlying structures/signals from noise. In PET (or SPECT) imaging, the raw data could be reconstructed using different parameters and settings, leading to different representations of the target image, which contain highly similar structures/signals to the target image contaminated with different noise levels (or properties). In this light, multiple-reconstruction NLM filtering (MR-NLM) is proposed, which relies on the redundant information provided by the different reconstructions of the same PET data (referred to as auxiliary images) to conduct the denoising process.

**Methods:**

Implementation of the MR-NLM approach involved the use of twelve auxiliary PET images (in addition to the target image) reconstructed using the same iterative reconstruction algorithm with different numbers of iterations and subsets. For each target voxel, the patches of voxels at the same location are extracted from the auxiliary PET images based on which the NLM denoising process is conducted. Through this, the exhaustive search scheme performed in the conventional NLM method to find similar patches of voxels is bypassed. The performance evaluation of the MR-NLM filter was carried out against the conventional NLM, Gaussian and bilateral post-reconstruction approaches using the experimental Jaszczak phantom and 25 whole-body PET/CT clinical studies.

**Results:**

The signal-to-noise ratio (SNR) in the experimental Jaszczak phantom study improved from 25.1 when using Gaussian filtering to 27.9 and 28.8 when the conventional NLM and MR-NLM methods were applied (*p* value < 0.05), respectively. Conversely, the Gaussian filter led to quantification bias of 35.4%, while NLM and MR-NLM approaches resulted in a bias of 32.0% and 31.1% (*p* value < 0.05), respectively. The clinical studies further confirm the superior performance of the MR-NLM method, wherein the quantitative bias measured in malignant lesions (hot spots) decreased from − 12.3 ± 2.3% when using the Gaussian filter to − 3.5 ± 1.3% and − 2.2 ± 1.2% when using the NLM and MR-NLM approaches (*p* value < 0.05), respectively.

**Conclusion:**

The MR-NLM approach exhibited promising performance in terms of noise suppression and signal preservation for PET images, thus translating into higher SNR compared to the conventional NLM approach. Despite the promising performance of the MR-NLM approach, the additional computational burden owing to the requirement of multiple PET reconstruction still needs to be addressed.

## Introduction

Positron emission tomography (PET) images commonly suffer from high level of noise, which hampers their clinical value [1, 2]. Statistical iterative reconstruction algorithms, including maximum likelihood expectation maximization (MLEM) and ordered subset-expectation maximization (OSEM) attempt to model the physical degradation factors to enhance the quality and quantitative accuracy of PET images. However, due to the inherent ill-posedness of the reconstruction problem, achieving full convergence, while avoiding noise amplification at the same time is challenging [3, 4].

A common strategy adopted to reduce noise in PET images is post-reconstruction filtering (usually Gaussian smoothing) prior to quantitative analysis and/or clinical interpretation [5, 6]. Noise reduction in PET imaging using post-reconstruction approaches commonly causes loss of significant signal and/or quantitative bias. In this regard, edge-preserving denoising approaches, which attempt to achieve effective noise reduction with minimal quantitative bias, were proposed to enhance the signal-to-noise ratio (SNR) in PET images [7]. Commonly used edge-preserving denoising approaches include bilateral and non-local mean in the image domain [7, 8] and wavelet or curvelet-based filters in the transform domain [9, 10].

Among the edge-preserving denoising techniques, the non-local mean approach (NLM) [11] has exhibited promising performance for the task of noise reduction while preserving significant PET signals or underlying structures [5, 12, 13]. The fundamental idea behind the NLM denoising approach is to explore the non-local areas within the image (could be at any distance from the target voxel/patch) to find similar patterns or textures. NLM filters rely on these forms of redundant information to effectively discriminate the genuine signal from the unwanted noise. An essential factor that impacts the performance of the NLM approach is the effectiveness of the patch search scheme to find and extract similar patterns (to the target patch) within the image. In this regard, different schemes of patch search have been proposed to conduct an effective search to find similar patterns and textures, thereby enhancing the quality of the NLM denoising approach in PET imaging [5, 8, 13].

This work sets out to introduce a novel patch search scheme for the NLM denoising approach particularly applicable to PET and single-photon emission tomography (SPECT) imaging. In the conventional NLM approach, a subset of the target image (or the entire image) is explored to find a number of similar patches, thereby conducting the denoising process for the target patch. The proposed algorithm relies on the fact that PET (or SPECT) raw data can be reconstructed using different numbers of iterations and subsets when iterative reconstruction algorithms are used. In this light, different representations of the target image (reconstructed using conventional/standard reconstruction algorithm and settings) could be generated using different reconstruction settings that highly resemble the target image. These accessory images consist of the same underlying structures/textures to the target image contaminated with different patterns or levels of noise. Therefore, these images enable to supply the NLM approach with highly similar (ideal) patches to conduct the denoising process on the target image.

In this work, we set out to examine the feasibility of using multiple PET image reconstructions to guide the NLM approach (so-called MR-NLM) for the task of noise reduction in whole-body PET imaging. Contrary to conventional NLM, wherein the search for similar patches is conducted within the same image, MR-NLM conducts the patch search across different reconstructed images of the same PET data. The proposed MR-NLM algorithm is evaluated against the conventional NLM approach, bilateral and Gaussian filters using experimental phantom and clinical whole-body PET/CT studies.

## Materials and methods

### Multiple-reconstruction non-local mean (MR-NLM) filter

**Fig. 1:**
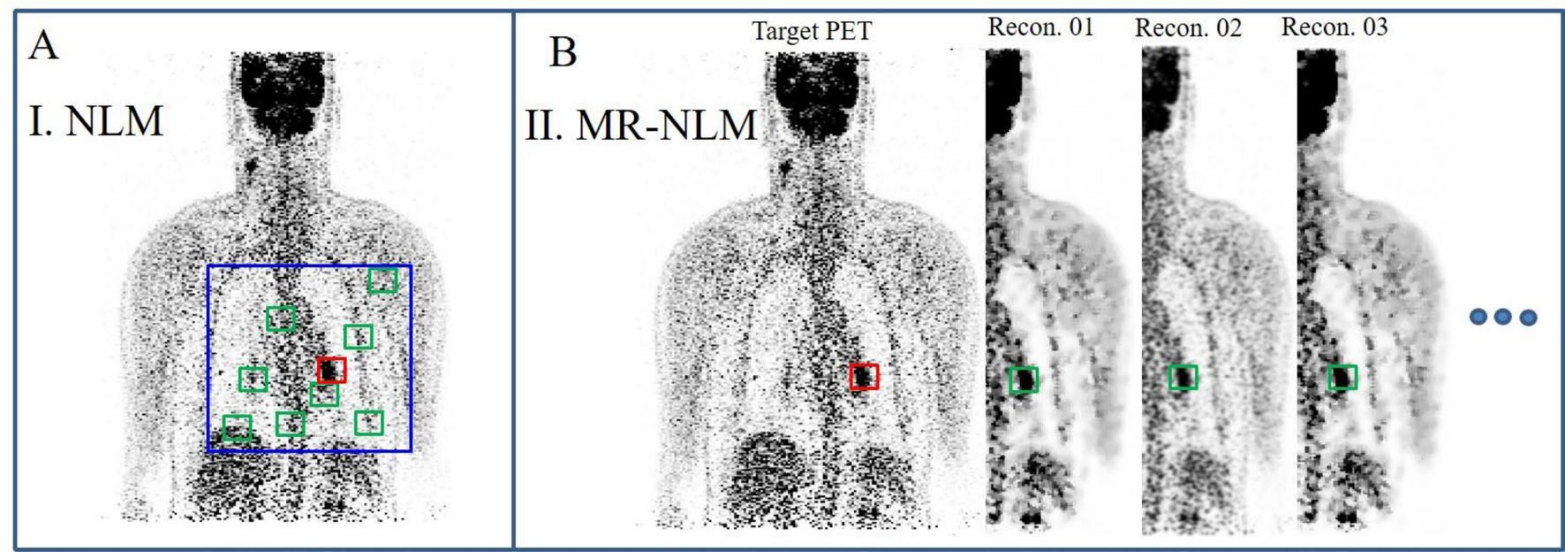
**a** Conventional non-local mean filter, wherein the search window, target patch and similar patches are indicated in blue, red and green squares, respectively. **b** Multiple-reconstruction NLM filter (MR-NLM) and similar patches of voxels across different reconstructions of the PET data

The NLM filter relies on the redundant information existing in the target image in the form of repeated textures/patterns or symmetrical structures. The NLM filter seeks to find similar patches of voxels within the image to suppress the noise through taking the weighted average (based on similarity to the target patch of voxels) of the selected patches. Finding similar patches is the key factor determining the performance of the NLM filter, which is conventionally carried out within a predefined search window (Fig. 1a). Given a number of patches of voxels, the denoising of the target image is conducted through employing Eqs. 1–5, wherein *V* denotes the output (denoised image), *f* is the noisy image (target image), *V*_*n*_ and *f*_*m*_ represent the *n*th and *m*th elements (voxel indices) of the target image before and after denoising, respectively. *w (n,m)* indicates the degree of similarity between two patches of voxels, *v*_*n*_ and *v*_*m*_, and *N*_*n*_ denotes a normalization factor to define the range of *w*. $\left| v \right|$ and Ω indicate the patch size and the size of the search window. *f*_*m(k)*_ indicates the *m*th element of the image *f*, which also belongs to the patch *v* with index *k* within the patch. *h* is the free parameter determining the level of smoothness:

1 $$V_{n} = \sum\nolimits_{{m \in \Omega _{n} }} {f_{m} ~w\left( {n,m} \right)} ,$$

2 $$w\left( {n,m} \right) = ~\frac{1}{{N_{n} }}\exp \left( { - \frac{{\left\| {P\left( {v_{n} } \right) - P\left( {v_{m} } \right)} \right\|^{2} }}{{h^{2} }}} \right),$$

3 $$P\left( {v_{n} } \right) = \left( {f_{n} , n \in v_{n} } \right),$$

4 $$\left\| {P\left( {v_{n} } \right) - P\left( {v_{m} } \right)} \right\|^{2} = \sum\nolimits_{{k = 1}}^{|v|} {\left( {f_{{n\left( k \right)}} - f_{{m\left( k \right)}} } \right)^{2} },$$

5 $$N_{n} = \sum\nolimits_{m} {w\left( {n,m} \right)} = \sum\nolimits_{m} {\exp \left( { - \frac{{\left\| {P\left( {v_{n} } \right) - P\left( {v_{m} } \right)} \right\|^{2} }}{{h^{2} }}} \right)} .$$

Essentially, the effectiveness of the NLM filter depends on the process of similar patch finding and to what extent the selected patches bear similar signal/texture/pattern to the target patches of voxels. As such, a strategy that provides to the NLM filter highly similar patches of voxels can greatly improve the performance of this approach. Since raw PET (or SPECT) data, in the list mode or sinogram formats, can be reconstructed several times with slightly different settings/parameters (even using different reconstruction algorithms), representations of the PET data or highly similar images to the target PET image can be generated for use within the NLM denoising approach. These representations of the PET data bear almost the same PET signals/structures contaminated with different noise patterns or structures, providing an ideal input for the NLM filter. Given the different reconstructions of PET data, the NLM filter would able to explore these images (at the same location of the target patch) to select/utilize similar patches of voxels (Fig. 1b). In this version of the NLM filter, referred to as multiple-reconstruction-NLM (MR-NLM), the search window (Ω) in the conventional NLM filter (blue box in Fig. 1a) would be replaced by a number of reconstructions of the PET data, wherein each reconstruction provides a single patch to the core of the MR-NLM filter. Apart from the procedure to select/find similar patches of voxels, the rest of the MR-NLM filter is the same as the conventional NLM formulated in Eq. 1.

### Algorithmic implementation

**Fig. 2:**
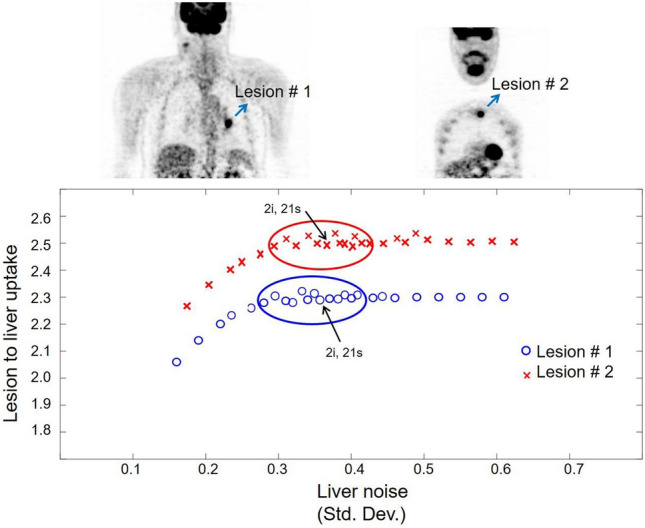
Lesion-to-liver uptake ratio versus the noise in the liver (standard deviation) for two malignant lesions of clinical PET/CT studies. Each small circle or cross indicates iteration/subset pairs used for the reconstruction of PET images. The target PET images were reconstructed using 2 iterations and 21 subsets (2i, 21s). The selected iteration/subset pairs for implementation of the MR-NLM filter includes 2i:24s, 2i:28s, 3i:14s, 3i:12s, 3i:21s, 4i:12s, 4i:8s, 5i:8s, 5i:7s, 5i:6s, 6i:7s, and 7i:6s. These pairs are enclosed in the blue and red circles

To implement the MR-NLM filter, the raw PET data should be reconstructed several times with different parameters/settings to generate various representations of the PET data (auxiliary PET images) bearing different noise levels, convergence and/or signal-to-background contrast. The target PET image should be reconstructed using the default reconstruction and hyperparameters. In this work, the target PET images were reconstructed using the standard parameters used in clinical setting, that is, TOF/PSF OP-OSEM (time-of-flight/point spread function ordinary Poisson ordered subset-expectation maximization) algorithm with 2 iteration and 21 subsets. To generate auxiliary PET images, different reconstruction algorithms, for instance, including filtered backprojection, MLEM or OSEM, could be employed. In addition, different versions of the same reconstruction algorithm, such as OP-OSEM without TOF data or PSF modeling could be exploited to generate auxiliary PET images. We set out to implement the MR-NLM filter using the same reconstruction algorithm (TOF/PSF OP-OSEM) but with different numbers of iterations and subsets to generate auxiliary PET images. To this end, the numbers of iterations and subsets should not be selected randomly, since they may result in dramatically different noise levels or poor convergence properties. In this light, iteration/subset pairs were selected in such a way to lead to similar convergence (signal-to-background ratio) and noise levels to the target PET images. PET image reconstruction was repeated several times using different iteration/subset pairs. Thereafter, 12 pairs were selected, which resulted in similar performance. The estimated noise levels in the liver and the lesion-to-liver uptake ratio in 8 clinical PET/CT studies were examined to select these 12 pairs. Figure 2 depicts two clinical PET studies, wherein the lesion-to-liver uptake ratios are plotted against the noise levels in the liver (standard deviation) for two malignant lesions. The raw PET data were reconstructed using various iteration and subset numbers to plot lesion contrast versus noise. The target PET images were reconstructed using 2 iterations and 21 subsets, resulting in an effective iteration number of 42 (iterations × subsets) [14]. To generate auxiliary PET images, iteration/subset pairs were initially examined to yield effective iteration numbers close to the target PET image (2 × 21). Among these pairs, 12 pairs of iteration and subset numbers were selected for final implementation of the MR-NLM filter, which had the closest performance to the target image in terms of lesion contrast versus noise, as depicted in Fig. 2. The target PET image is indicated by an arrow and the selected 12 iteration/subset pairs are enclosed in a circle. These 12 pairs include 2i:24 s (2 iterations and 24 subsets), 2i:28 s, 3i:14 s, 3i:12 s, 3i:21 s, 4i:12 s, 4i:8 s, 5i:8 s, 5i:7 s, 5i:6 s, 6i:7 s, and 7i:6 s. It should be noted that the Biograph mCT scanner creates PET sinograms with 168 angular samples or bins, thus the number of subsets should be a divisor of 168. For this reason, these specific subset numbers are employed, for instance, subset number of 22 could not be used as it is not a divisor of 168. The Biograph mCT provides a TOF resolution of 530 ps split into 13 TOF bins, with each bin 312 ps wide. The PET data in the sinogram space for each bed position consist of 400 (bins) × 168 (angles) × 621 (planes in 9 segments) × 13 (TOF bins).

### Experimental and clinical studies

The proposed MR-NLM approach for denoising of PET images was investigated against the conventional NLM, bilateral, and commonly used post-reconstruction Gaussian filters. The assessment of these denoising approaches was carried out using experimental phantom and clinical whole-body PET/CT studies.

For the experimental phantom study, the physical Jaszczak phantom consisting of six spheres with diameters of 11.89, 14.43, 17.69, 21.79, 26.82 and 33.27 mm inserted within a cylindrical container with a radius of 100 mm and height of 180 mm. The cylindrical container, referred to as background medium, was filled with an activity concentration of 3.6 kBq/ml. The six spherical inserts were filled with an activity of 18.4 kBq/ml to create a 5:1 signal-to-background contrast. PET/CT data acquisition was performed on a Biograph mCT PET/CT scanner (Siemens Healthcare, Knoxville, TN) for a total of 30 min. The raw PET data were then reconstructed for time frames of 10 s, 30 s, 1 min, 3 min, 10 min, and 30 min, thereby creating six images of the phantom with six different noise levels. PET image reconstruction was performed using OP-OSEM algorithm (2 iterations, 21 subsets, matrix of 400 × 400 × 168–254 and voxel size of 2 × 2 × 3 mm) with TOF and PSF modeling using the e7 tool (Siemens Healthcare, Knoxville, TN). PET image reconstruction included CT-based scatter and attenuation corrections.

For the clinical studies, we exploited the raw PET data of twenty-five whole-body PET/CT studies (mean age ± SD = 61 ± 7 years and mean weight ± SD = 69 ± 9 kg) performed on the same Biograph mCT PET/CT scanner 60 min post-injection of 251.5 ± 44.5 MBq of ^18^F-FDG. The average acquisition time was 25.6 ± 5.5 min considering continuous bed speed of 0.7 mm/s. A whole-body CT scan was performed using 150 mAs, 110–120 kVp, and slice thickness of 5 mm was performed for attenuation correction. The clinical PET image reconstructions were performed using TOF/PSF OP-OSEM algorithm with 2 iterations, 21 subsets, a matrix of 200 × 200, and voxel size of 4 × 4 × 2 mm. The conventional post-reconstruction Gaussian filtering, applied by default in the clinic, was deactivated to obtain unfiltered (noisy) PET images.

To assess the performance of the different denoising approaches, 50 volumes of interest (VOIs) were manually drawn on malignant lesions and high uptake regions. Nineteen VOIs were in soft-tissue, 16 near bony structures, and 15 in the lung region with volumes ranging between 0.5 and 2.0 ml. To obtain the corresponding background VOIs for calculation of the lesion-to-background contrast, VOIs with the same size and shape were defined next to each lesion in the same background region. The high uptake regions and malignant lesions were located in different regions of the body bearing various lesion-to-background contrasts. Moreover, the liver and lung regions were delineated on CT images of 20 patients to estimate the mean and standard deviation of the radiotracer concentration within these regions before and after the application of the denoising approaches.

### Quantitative analysis

The MR-NLM approach was evaluated against the conventional NLM, bilateral filter [7] and commonly used Gaussian post-reconstruction denoising methods. The kernel of the Gaussian filter varied from 2 to 9 mm in terms of full-width half-maximum (FWHM) for the physical Jaszczak phantom study depending on the acquisition times or noise levels. Similarly, in the clinical studies, kernels of 2–6 mm (FWHM) were used for Gaussian filtering. It should be noted that a set of smoothing parameters may not be optimal for all phantom and clinical studies. As such, depending on the level of noise in the input images, the smoothing parameters (such as FWHM for the Gaussian filter or free smoothing parameter h for the NLM filter) were slightly modified to achieve the highest SNR in the resulting images. The results averaged over all input images and/or noise levels are reported the results section.

The bilateral filter consists of two separate Gaussian kernels in the spatial and intensity domains regulated by *σ*_Sp_ and *σ*_In_ free parameters, respectively [15]. The range of *σ*_In_ varied from 0.2 to 0.5 depending on the noise levels of the images, whereas *σ*_Sp_ was set to 3.8 as recommended in [7].

For the experimental phantom and clinical studies, the contrast-to-noise ratio (CNR) (defined in Eq. 6) was estimated for the spheres in the Jaszczak phantom before and after application of the different denoising techniques. PET images obtained without denoising (without any post-reconstruction filtering) is referred to as OSEM:

6 $${\text{CNR}} = \frac{{\left| {\mu_{{{\text{signal}}}} - \mu_{{{\text{bg}}}} } \right|}}{{\sqrt {\frac{{\left| {\sigma_{{{\text{signal}}}}^{2} + \sigma_{{{\text{bg}}}}^{2} } \right|}}{2}} }},$$

where *μ*_signal_ and *μ*_bg_ denote the mean values of the voxel intensities in the target and background VOIs, respectively. The corresponding standard deviation of the target and background VOIs are indicated by *σ*^*2*^_signal_ and *σ*^*2*^_bg_, respectively. In addition, the SNR and quantitative bias (%) were estimated for the hot spheres of the Jaszczak phantom as well as the VOIs drawn on the clinical PET studies using Eqs. 7 and 8, respectively:

7 $${\text{SNR}} = \frac{1}{{{\text{Ns}}}}\sum\nolimits_{k = 1}^{{{\text{Ns}}}} {\frac{{\left( {\mu_{{{\text{signal}}\left( k \right)}} - \mu_{{{\text{bg}}\left( k \right)}} } \right)}}{{\sigma_{k} }}} ,$$

8 $${\text{Bias}}_{{{\text{phantom}}}} \left( \% \right) = 100 \times \frac{{\mu_{{{\text{signal}}}} - \mu_{{{\text{reference}}}} }}{{\mu_{{{\text{reference}}}} }}.$$

In Eq. 7, Ns denotes the number of VOIs across all patients, whereas *σ*_*k*_ stands for the standard deviation within the VOIs drawn on the background. Equation 8 was used for the calculation of quantification bias in the experimental phantom study, since the activity concentration within the VOIs is known (reference). Since the actual activity concentration is not known in clinical studies, the quantitative bias resulting from the different denoising approaches was calculated against the unfiltered (noisy) PET images using the following equation:

9 $${\text{Bias}}_{{{\text{patient}}}} \left( \% \right) = 100 \times \frac{{\left| {\mu_{{{\text{signal}}}} - \mu_{{{\text{noisy}}}} } \right|}}{{\mu_{{{\text{noisy}}}} }},$$

where *μ*_noisy_ indicates the mean activity concentration in VOIs drawn on the unfiltered noisy PET images. The statistical significance of the differences between the quantitative metrics derived from the conventional NLM and MR-NLM filters were assessed using the paired *t* test method considering a *p* value threshold of 0.05 as threshold of statistical significance.

The patch size (*v*_*m*_ or *v*_*n*_) and the free parameter *h* (determining the levels of smoothness) in Eq. 5 are the key parameters to be optimized to reach the peak performance of the MR-NLM denoising approach. To this end, different values were assigned to these free parameters to achieve the highest contrast-to–noise ratio (CNR) and signal-to-noise ratio (SNR) in the experimental phantom and clinical studies. Regarding *v*_*m*_ or *v*_*n*_ parameters, a patch size of 5 × 5 voxels led to the best performance for both phantom and clinical studies. The smoothing parameter *h* depended on the levels of noise in the input images. For the clinical study, the optimum value of this parameter was found (*h* = 1.5 × 10^–3^), while for the phantom study, depending on the level of noise in the input images, the optimal *h* value varied between 10^–3^ and 2 × 10^–3^ for low-noise to high-noise levels, respectively.

Given a number of auxiliary PET images obtained from multiple reconstructions of the PET data, a comparison of the weighted average (MR-NLM) versus mean of these auxiliary images (non-weighted) would demonstrate the efficiency/necessity of weight factors calculation for weighted averaging. In this regard, CNR, SNR, and quantification bias (Eq. 8) were calculated and compared for these two approaches over the six spheres of the Jaszczack phantom.

## Results

**Fig. 3:**
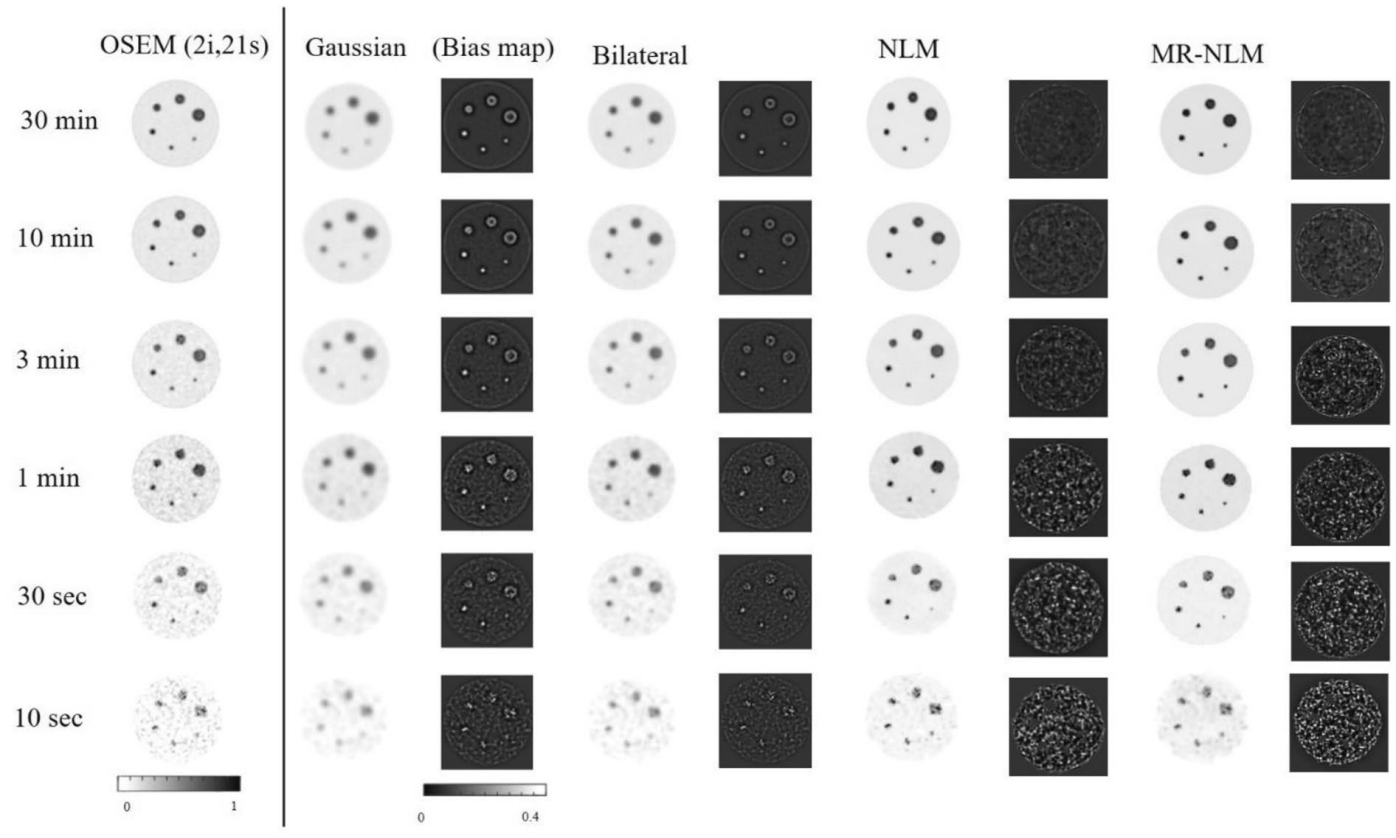
Reconstructed images of the Jaszczack phantom for the different acquisition times. The first column shows the unfiltered images (OSEM) compared to images filtered with the Gaussian, bilateral, NLM, and MR-NLM approaches. The corresponding bias maps (OSEM-filtered image) are also shown

Figure 3 depicts slices of the Jaszczak phantom reconstructed using different acquisition times, namely 30 min, 10 min, 3 min, 1 min, 30 s, and 10 s, presenting different noise levels. The images of the phantom are shown before (OSEM) and after application of the post-reconstruction filters along with the corresponding bias map. Visual inspection revealed that the MR-NLM approach led to overall more effective noise suppression and less resolution/signal loss, while the conventional NLM method exhibited close performance.

**Fig. 4:**
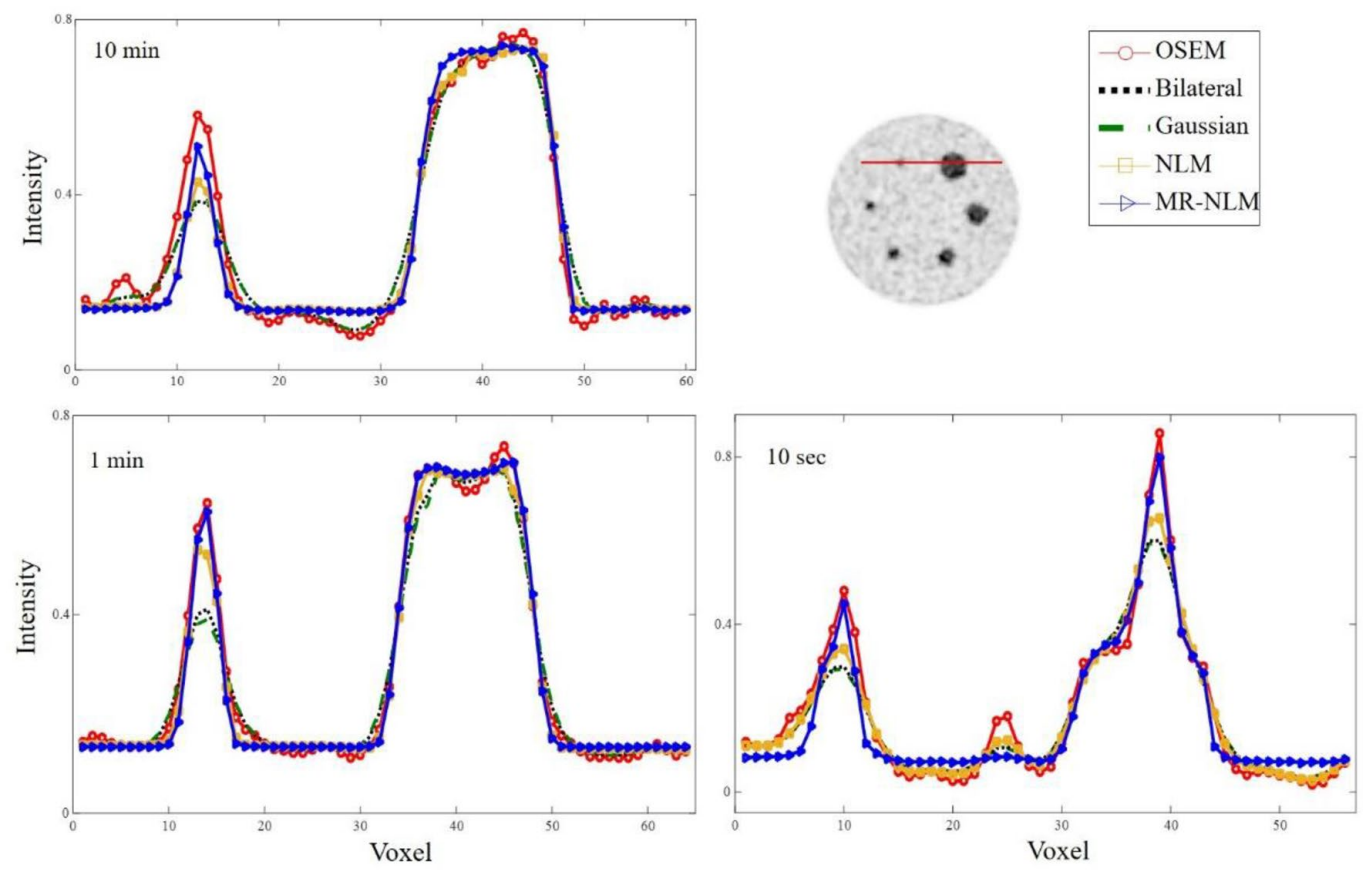
Horizontally profiles drawn images of the Jaszczack phantom before (OSEM) and after application of the different post-reconstruction filters. The profiles are drawn across the smallest and largest spheres with diameters of 11.89 mm and 33.27 mm, respectively

Figure 4 illustrates the profiles plotted horizontally across the two spheres (smallest and largest) of the Jaszczack phantom. MR-NLM exhibited less signal/resolution loss, particularly when considering the plot over the small sphere, wherein other methods yielded noticeable signal loss.

The CNR, SNR and quantification bias estimated for the six spheres of the Jaszczak phantom (from smallest #1 to largest #6) are reported in Table 1. Gaussian filtering resulted in the largest bias observed (> 35%) in the smallest sphere, while NLM and MR-NLM yielded biases of 32.0% and 31.1% (*p* value < 0.05) for the same sphere, respectively. The *p* values reported in Table 1 were calculated between the NLM and MR-NLM approaches. In the phantom study, the bias for the different approaches was calculated using the actual activity concentration within the spheres and the background. Hence, OSEM reconstruction of the phantom already bears a bias of 29.2% for the smallest sphere.

**Table 1:** Contrast-to-noise ratio (CNR), signal-to-noise ratio (SNR) and quantification bias (based on Eq. 8) calculated for spheres #1–#6 corresponding to the smallest to the largest spheres in the Jaszczak phantom

Sphere	#1	#2	#3	#4	#5	#6
OSEM						
Bias (%)	29.2	27.4	25.2	25.1	24.3	22.3
SNR	20.2	21.0	21.7	22.6	23.0	22.8
CNR	5.9	6.2	8.2	9.1	8.2	9.1
Gaussian						
Bias (%)	35.4	32.6	30.7	28.5	27.0	24.7
SNR	25.1	25.9	26.4	26.9	27.3	27.2
CNR	6.3	8.4	11.3	14.3	14.1	13.5
Bilateral						
Bias (%)	33.3	31.3	28.6	27.4	26.2	24.3
SNR	26.0	26.3	27.8	28.0	28.4	28.4
CNR	8.8	10.0	15.1	17.5	17.7	18.3
NLM						
Bias (%)	32.0	30.9	28.1	27.2	25.9	23.9
SNR	27.9	28.3	29.1	31.3	31.4	31.5
CNR	12.9	13.1	17.8	21.6	22.1	24.1
MR-NLM						
Bias (%)	31.1	29.7	29.0	26.6	25.1	23.1
SNR	28.8	29.9	30.1	32.5	32.6	32.7
CNR	13.8	13.9	18.8	22.6	22.9	24.9
*p* value						
Bias (%)	0.04	0.03	0.01	0.04	0.04	0.05
SNR	0.02	0.01	0.02	0.01	0.03	0.04
CNR	0.03	0.05	0.02	0.03	0.05	0.06

Overall, Gaussian filtering resulted in poor SNR for all six spheres with the highest value of 27.3, while the NLM and MR-NLM approaches led to the SNRs of 31.5 and 32.5 (in the largest sphere), respectively.

**Fig. 5:**
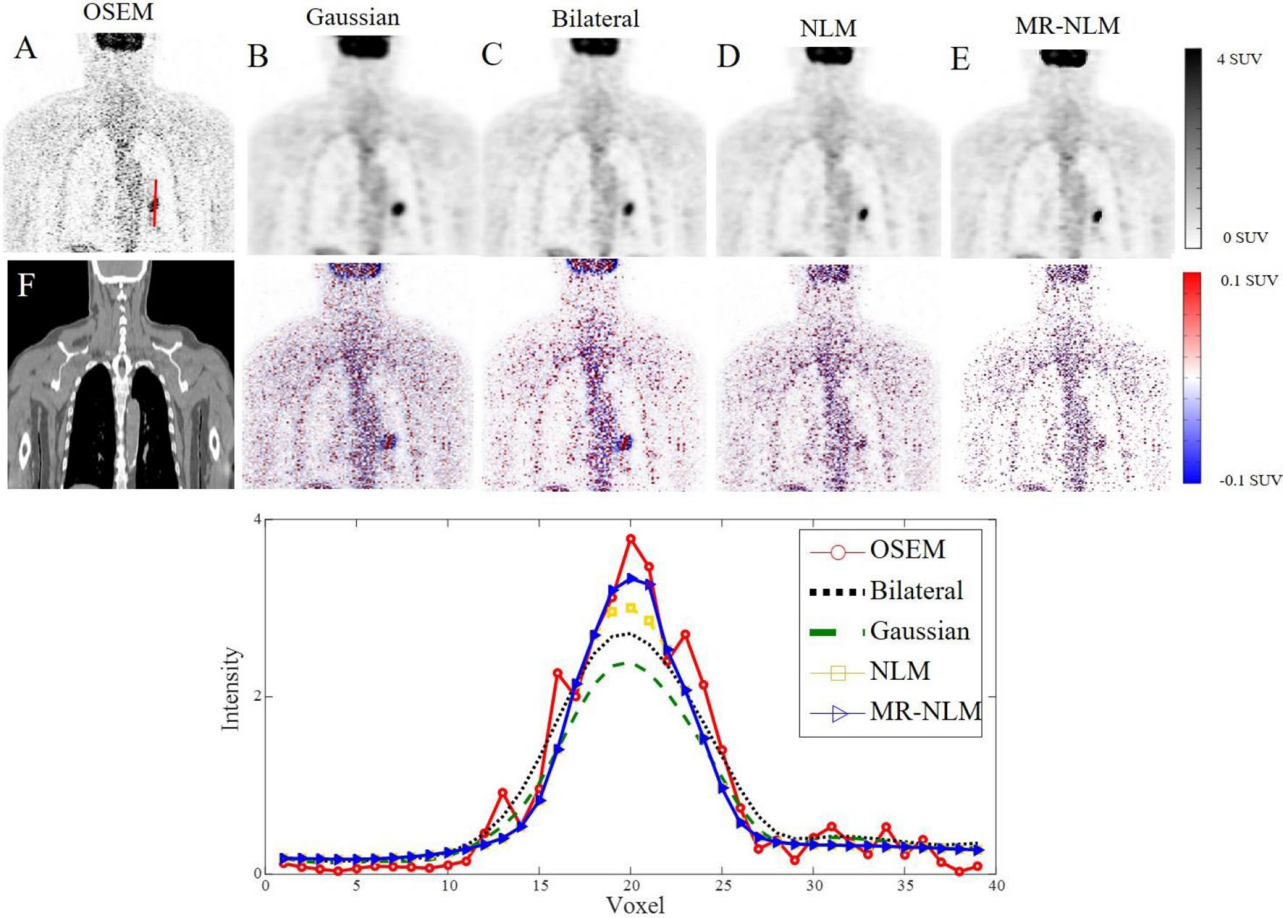
Representative coronal views of PET and CT images of a patient presenting with non-small lung cancer. **a** Original unfiltered (OSEM) PET image and filtered using, **b** Gaussian, **c** bilateral, **d** NLM and **e** MR-NLM filters. The corresponding bias map (filtered—OSEM) is also displayed below each image. **f** The corresponding CT image. The vertical line profile plotted over the lung lesion is illustrated in the bottom panel

Figure 5 depicts transaxial views of clinical PET images of a patient presenting with non-small lung cancer before and after application of the different denoising approaches. Visual inspection revealed over-smoothed structures when using Gaussian filtering compared to the NLM and MR-NLM filtered images, wherein less signal/resolution loss and effective noise suppression are observed. The unfiltered PET image (OSEM) (Fig. 5a) did not undergo any post-reconstruction filtering. Considering the bias maps (filtered—OSEM), Gaussian and MR-NLM filters led to the largest and smallest signal loss, respectively, particularly for the small lesion in the lung. The vertical profile plotted on the small lung lesion demonstrates effective denoising and minimal signal loss when using the MR-NLM approach in comparison to Gaussian and bilateral filtering with noticeable signal loss.

Table 2 presents the quantitative evaluation of the different denoising approaches on the clinical whole-body PET/CT studies. The MR-NLM algorithm led to an overall of bias and SNR of − 2.2 ± 1.2% and 34.9 ± 5.7, respectively, for the high uptake regions and malignant lesions, while the conventional NLM approach resulted in bias and SNR of − 3.5 ± 1.3% and 32.4 ± 5.5 (*p* value < 0.02), respectively, demonstrating superior performance of the MR-NLM algorithm. It should be noted that the biases reported in Table 2 were calculated against unfiltered (OSEM) PET images using Eq. 9.

**Table 2:** Contrast-to-noise ratio (CNR), signal-to-noise ratio (SNR) and quantitative bias (based on Eq. 9) (± standard deviation) calculated against the unfiltered (OSEM) PET images in the clinical studies

	CNR	SNR	Lesion SUV_mean_ (Std. Dev.)	Lung SUV_mean_ (Std. Dev.)	Liver SUV_mean_ (Std. Dev.)	Bias (%) (for lesions)
OSEM	13.2 ± 3.7	23.3 ± 6.8	7.8 ± 2.0 (1.6)	0.29 ± 0.08 (0.098)	2.99 ± 0.8 (0.32)	–
Gaussian	14.9 ± 3.6	26.5 ± 5.5	6.6 ± 1.9 (1.0)	0.25 ± 0.07 (0.075)	2.80 ± 0.7 (0.27)	− 12.3 ± 2.3
Bilateral	18.9 ± 4.1	29.2 ± 5.9	6.8 ± 1.9 (1.1)	0.25 ± 0.06 (0.077)	2.89 ± 0.7 (0.26)	− 7.7 ± 2.1
NLM	23.4 ± 4.4	32.4 ± 5.5	7.1 ± 1.7 (0.8)	0.26 ± 0.05 (0.052)	2.90 ± 0.7 (0.22)	− 3.5 ± 1.3
MR-NLM	25.0 ± 4.0	34.9 ± 5.7	7.3 ± 1.6 (0.7)	0.27 ± 0.04 (0.050)	2.94 ± 0.6 (0.19)	− 2.2 ± 1.2
*p* value	0.01	0.02	0.03	0.05	0.04	0.02

Mean SUV estimated in the liver, lung, and malignant lesions within these regions are also reported

Table 3 compares the performance of the MR-NLM approach with the mean of the auxiliary images (non-weighted) over the six spheres of the Jaszczack phantom. Statistically significant improvement (*p* value < 0.01) was observed when the weighted average of the auxiliary images was considered compared to simple averaging.

**Table 3:** Contrast-to-noise ratio (CNR), signal-to-noise ratio (SNR) and quantification bias (based on Eq. 8) calculated for images obtained from simple averaging of all auxiliary images (MR-average) and the MR-NLM method

Sphere	#1	#2	#3	#4	#5	#6
MR-average						
Bias (%)	32.1	31.1	29.9	27.2	26.0	23.4
SNR	27.7	28.0	28.8	31.0	30.4	30.7
CNR	12.9	12.9	17.7	21.5	21.7	23.9
MR-NLM						
Bias (%)	31.1	29.7	29.0	26.6	25.1	23.1
SNR	28.8	29.9	30.1	32.5	32.6	32.7
CNR	13.8	13.9	18.8	22.6	22.9	24.9

## Discussion

This work introduced multi-reconstruction non-local mean filter as a variant of the well-established NLM denoising approach dedicated for denoising of PET and SPECT images but is applicable to any other modality, where the raw data can be reconstructed/reformatted to generate different noise realizations of the same image. The MR-NLM approach relies on the redundant information which could be generated from the same raw data, wherein the underlying signals/structures are represented with different amount of noise and slightly different signal-to-background contrast. In the conventional NLM approach, a search scheme should be implemented to find similar patches of the voxel within the same image to conduct the denoising process of the target patch of voxels [8, 13]. Intuitively, the performance of NLM filtering depends on the level of redundant information present in the target image. This solution is appropriate for most natural images, since they normally contain sufficient repeated structures, patterns and symmetries. Moreover, for natural images, a single version of the image exists and regeneration of the same image with different noise realization is not easily feasible. Conversely, in PET and SPECT imaging, though sufficient redundant information might exist within the target image, highly similar information could be generated through reconstruction of the raw data to effectively conduct the NLM denoising process. In the MR-NLM approach, due to the presence of sufficient redundant information across the auxiliary images (different reconstructed images), there is no need for a search window, which is defined in the conventional NLM approach to restrict the search for similar patches to a specific local neighborhood.

PET image reconstruction could be carried out using different reconstruction algorithms, namely filter backprojection, MLEM, attenuation weighted OSEM, etc. combined with or without PSF modeling. Moreover, PET scanners with TOF capability allow for reconstruction of PET images with and without TOF information. Each of the abovementioned reconstruction algorithms would lead to different convergence, contrast and noise characteristics. Investigation of all reconstruction algorithms and their benefits on MR-NLM filtering warrants a separate study. In this work, a standard reconstruction algorithm used in clinical practice (OP-OSEM with TOF/PSF) was investigated, wherein the auxiliary PET images were generated through varying the iteration and subset numbers. Varying the effective number of iterations (iteration × subset) would impact the convergence of image reconstruction as well as the noise properties of the image. As such, given the standard number of iterations and subsets (2i and 21s), several effective iteration numbers close to the standard one (2 × 21) were examined. Auxiliary images with highly increased noise levels or poor convergence (signal-to-background contrast) were not beneficial to the MR-NLM denoising process. Conversely, auxiliary PET images with similar noise properties and convergence to the target images contribute effectively to the denoising process. Therefore, the twelve (in addition to target image) closest auxiliary PET images were selected (as indicated in Fig. 2) to implement the MR-NLM denoising approach. Incorporating more than twelve auxiliary images did not significantly improve the performance of the MR-NLM filter.

Given a number of auxiliary images needed to implement the MR-NLM, taking the mean of auxiliary images (instead of conducting weighted averaging) might also result in satisfactory outcome. To investigate this alternative, a simple average of all auxiliary images (including the target image) was performed for the phantom study and the associated results are reported in Table 3. Comparing the results in Table 3 with NLM in Table 1 showed that a simple average of all auxiliary images would lead to suboptimal denoising outcome with slightly higher bias and no improvement in SNR.

The quantitative evaluation conducted on the experimental Jaszczak phantom and clinical whole-body PET/CT studies exhibited the promising performance of the MR-NLM algorithm versus the conventional NLM approach. Enhanced SNR along with reduced signal loss were achieved by MR-NLM filtering in comparison to the NLM method in both phantom and clinical studies. The promising performance of the MR-NLM algorithm results from the presence of highly similar patches of voxels across auxiliary PET images, which might not be found within the search window of the conventional NLM filter.

In addition to the conventional NLM filter, post-reconstruction bilateral and Gaussian filters were evaluated in this study in an attempt to provide a broader view over the performance of the MR-NLM technique. Considering the various quantitative metrics, MR-NLM outperformed the other denoising approaches in both phantom and clinical studies leading to significantly less signal loss and enhanced SNR. Post-reconstruction Gaussian filtering is traditionally used in clinical practice to suppress noise and enhance the quality of PET images. Though this filter enables effective noise reduction, considerable signal loss and over-smoothed structures are inevitable which may adversely impact the quantitative potential of PET. Owing to the wide usage of this filter in clinical practice, this method is regarded as bottom line based on which the performance of the other denoising approaches was assessed. The MR-NLM approach demonstrated superior performance over Gaussian filtering in terms of signal preservation and effective noise reduction.

The performance of MR-NLM method was compared to the conventional NLM approach in terms of the key image quality metrics. However, the important issues of computational burden and processing time have not been discussed. The major limitation/pitfall of the MR-NLM approach is the requirement for multiple reconstructions (here 12) of PET data, which results in long processing time. In this regard, the computational time taken by the MR-NLM approach would be *N* times higher than the conventional NLM technique, where *N* is the number of required reconstruction of the PET data. It should be noted that regardless of the image reconstruction time, the MR-NLM filter is much faster than the NLM method, since there is no need to conduct a search to find similar patches (readily provided by auxiliary PET images) within the search window. Proportional to the size of the search window, the MR-NLM filter is faster than the NLM filter (the larger, the faster). However, the time required for multiple reconstructions of the PET data is notably higher than other processing techniques. A straightforward strategy to reduce the computational time is to use a single reconstruction with multiple save points. For instance, if reconstructions with 7i:6s are to be performed, the reconstructed images after 5 and 6 iterations could be saved, while the process continues to reach seven iterations. Moreover, since scatter estimation and correction is computationally intensive, a single scatter matrix (obtained from reconstruction of the target image with 2i:21s) could be employed for the reconstruction of all auxiliary images. These strategies could significantly reduce the processing time of the MR-NLM approach.

As a matter of fact, the performance of the NLM filter depends highly on the presence/detection of similar/repeated patches of voxels. In our previous study, a novel search scheme was proposed to aid the NLM smoothing approach to detect/find similar/repeated patterns [8]. This spatially guided-NLM (SG-NLM) approach enabled an effective exploration of the entire 3D image to maximize the detection of repeated/similar patches without significant additional processing time. SG-NLM exhibited enhanced performance over the conventional NLM filter, where the search for similar patches is only conducted in a limited area/volume around the target voxel. Since the SG-NLM filter explores the entire 3D image, there is a greater likelihood to find similar patches. Conversely, these similar patches are provided to the MR-NLM approach by the multiple image reconstructions. Hence, a comparable performance would be expected from these two approaches, though this claim warrants further comparison study. In terms of processing time, the SG-NLM is remarkably faster, because it does not require multiple reconstructions of the PET data. Nevertheless, there are instances, e.g., small lesions in the lung, where finding a sufficient number of similar patches may not be possible even through exploration of the entire 3D image. In these cases, the MR-NLM filter is expected to exhibit superior performance to other variations of the NLM filter. In this regard, a combination of the MR-NLM (for instance using fewer number auxiliary images) and SG-NLM approaches would lead to an optimal solution.

This study sets out to investigate the feasibility of using multiple reconstructions of the same PET data for noise suppression. Different reconstructions of the PET data contain different noise levels, properties or characteristics while keeping almost the same underlying signals/structures. The idea was to employ this information to enable the MR-NLM approach to discriminate between noise and genuine signals more effectively. The ultimate objective is to employ this concept within a deep learning framework [16] to enhance PET image quality (denoising). This could be achieved using either an unsupervised or supervised training scheme. In addition, this concept could also be employed in low-dose PET imaging to estimate/predict high quality/standard PET images [17].

## Conclusion

We introduced a multiple-reconstruction non-local mean (MR-NLM) filter as variant of the NLM denoising approach dedicated for denoising of PET and potentially SPECT images. MR-NLM relies on multiple reconstructions of PET data using different reconstruction settings to realize different versions of the target image with various noise properties. The conventional NLM approach requires an exhaustive search to find similar patches of voxels within the target image based on which the signal and noise discrimination is carried out. Since multiple PET reconstructions readily provides highly similar patches of voxels, MR-NLM does not require exhaustive patch search and is able to achieve noise suppression with higher accuracy. Experimental phantom and clinical studies demonstrated the superior noise suppression and signal preservation achieved by the MR-NLM approach in comparison to conventional NLM, Gaussian and bilateral denoising approaches. Despite the promising performance of the MR-NLM filter, implementation of this approach enforces high computational burden, since multiple PET reconstructions are required.
